# First case recognized as autoimmune polyglandular syndrome type 2 with myasthenia gravis in Palestine: A case report and literature review

**DOI:** 10.1016/j.amsu.2021.102575

**Published:** 2021-07-16

**Authors:** Yousef S. Abuzneid, Yasmine Yaghi, Arein Madia, Nataly Salhab, Naser Amro, Sadi A. Abukhalaf, Mohammad Kharraz

**Affiliations:** a Al-Quds University Faculty of Medicine, Jerusalem, Palestine; b An-Najah National University Hospital, Nablus, Palestine

**Keywords:** Myasthenia gravis, Autoimmune polyglandular syndrome (APS), Type 2, Case report

## Abstract

**Background:**

Myasthenia gravis is an organ specific autoimmune disorder that is potentially serious but treatable. It is characterized by fatigability of the voluntary muscles and weakness caused by antibodies against the nicotinic acetylcholine receptor (AChR) on the postsynaptic membrane at the neuromuscular junction.

Sometimes, and in very rare cases, it can be associated with other autoimmune conditions in a so called autoimmune polyglandular syndrome type 2, which consists mainly of autoimmune adrenal insufficiency (Addison's disease) with autoimmune thyroid disease and/or type 1 diabetes mellitus.

**Case presentation:**

We describe a case of a 47-year-old male patient presenting with weakness, difficulty swallowing (mainly liquids) and dysarthria. He was discovered to have low cortisol and TSH levels with high T4 and T3. These findings lead to the suspicion of a more complex disease process and through a thorough research of literature we discovered an association between myasthenia gravis and autoimmune polyglandular syndrome specifically type 2 which fits with our patients’ presentation.

**Conclusion:**

In any autoimmune disease, it is important to keep in mind associations and susceptibilities to other autoimmune processes and syndromes in order to reach a correct diagnosis and treatment preventing life threating events.

## Highlights

• Myasthenia gravis is an autoimmune disease that can be a manifested with other autoimmune disorders.
• Careful history taking, meticulous physical examination, and investigations are necessary.
• This syndrome should be kept in mind even its rarity.

## 1 Introduction

Myasthenia gravis is an autoimmune disorder with autoantibodies against the nicotinic acetylcholine receptor on the postsynaptic membrane at the neuromuscular junction.

It is characterized by fatigability of the voluntary muscles and weakness with a bimodal peak presentation in the third decade and in the sixth decade [1].

The incidence of MG ranges from 0.25 to 2.0 per 1,000,000 and because of the effective treatment strategies and normal life expectancy, the prevalence of MG has increased in recent years to about 72: 1,000,000 (range 15–179) with about 10% being children and adolescents [2]. This condition is probably undiagnosed in the very old population because muscle weakness is a common complaint [1].

In addition, there is an increased familial risk for MG. Siblings or first-grade relatives of affected patients have a risk of 4.5% for developing MG reflecting a profound genetic disposition for the disorder [2].

The pathogenesis, immunology and molecular biology of myasthenia gravis has greatly improved within the past three decades and now it is almost always possible to establish a diagnosis with the available tests. Nowadays, the modern treatment is also very successful, and the mortality is practically zero. However, there are still some topics that are not fully understood like the factors that contribute to chronic disease and the way to fully cure this condition [1].

The manifestation of Addison's disease with autoimmune thyroid disease and/or type 1 diabetes mellitus is known as autoimmune polyglandular syndrome type 2 (APS-2). This syndrome is associated with HLA-DR3 and/or HLA-DR4 haplotypes. It is an autosomal dominant disease that has many variable clinical expressions [[3], [4], [5]].

Presentation is usually in the third to fourth decades of life with a high prevalence in middle-aged women. It's a very rare disease during childhood [6].

APS-2 is most frequent seen as a clinical combination of Addison's disease and Hashimoto's disease. Adrenal insufficiency was reported in 5.3% of patients with Hashimoto's disease and two to 4% of autoimmune thyroid disease patients had concomitant celiac disease [5].

The work has been reported in line with the SCARE 2020 criteria [16].

## 2 Case presentation

A 47-year-old male patient from Gaza was referred to our hospital due to body weakness, dysphagia and dysarthria of 14 days duration.

He was in his usual state of health until two weeks before admission when he started to complain of proximal muscle weakness that was increasing in severity in addition to fatigue, difficulty in speech and chocking episodes mainly triggered by liquids.

In Gaza hospital the doctors performed a brain and whole spine MRI which showed no abnormality. Furthermore, a lumbar puncture was done with normal results.

Due to a progressive deterioration of his symptoms, as his weakness was so severe he was almost became quadriplegic, his case was referred to our hospital.

An NG tube was introduced due to his severe dysphagia and he was reevaluated with a thorough physical examination that showed: mild respiratory distress with a respiratory rate of 19 breaths/min, vitiligo over his entire body, weak gag reflex and nasal speech. The lower limb weakness was 1/5 bilaterally and the upper limb weakness was 2/5 with intact sensation and hyporeflexia.

An electromyography (EMG) and nerve conduction studies were also performed and showed a decrement in the muscle action-potential which lead to the suspicion of myasthenia gravis. Thus a serum anti-Ach receptor antibodies titer was done with positive results leading to the confirmation of this suspicion.

During the patients’ time of hospitalization and evaluation, it was discovered that his fasting cortisol levels and TSH levels were low (TSH was 0.35 mU/L) whereas the T3 and T4 were high.

Since the patient was found to have findings of three autoimmune diseases, autoimmune polyglandular syndrome was considered.

Six sessions of plasmapheresis were done. The patient was also prescribed prednisolone pyridostigmine, azathioprine, vitamin D and thyroxin.

After we started his treatment, the patient's muscle power started to improve (after the third session of plasmapheresis we could notice the improvement).

In addition, physiotherapy was recommended and after six sessions he was able to walk alone again, the NG tube was removed and he was started on oral feeding with a semi-solid diet.

He was discharged later after a good response to treatment with a good prognosis.

## 3 Discussion

Myasthenia gravis consist of a fluctuating fatigability and weakness affecting the ocular, bulbar and upper and lower limbs (in a proximal skeletal muscle group way). It can occur as an autoimmune disease with a characteristic immunogenic pattern or as a paraneoplastic syndrome associated with tumors of the thymus [2].

Impairment of central thymic and peripheral self-tolerance mechanisms in both cases is thought to favor an autoimmune CD4 T cell-mediated that causes activation of the B cells and the synthesis of pathogenic high-affinity autoantibodies of either the IgG1 and 3 or IgG4 subclass. These autoantibodies bind to the nicotinic acetylcholine receptor (AchR) itself, or muscle-specific tyrosine-kinase (MuSK), lipoprotein receptor-related protein 4 (LRP4) and agrin involved in clustering of AchRs within the postsynaptic membrane and structural maintenance of the neuromuscular synapse. This results in disturbance of neuromuscular transmission, which causes the clinical manifestation of the disease [[7], [8], [9], [10], [11]].

The symptoms of MG can be: ocular symptoms (the most frequent) that include ptosis, diplopia, doble vision if it affects the extraocular muscles and in a minority of people can cause blurred vision, involvement of bulbar muscles which include those innervated by motor neurons originating from the pons and medulla oblongata are the ones responsible for difficulties in speech, nasal voice, dysphagia and later on difficulties in chewing and being able to open the mouth, weakness of neck and facial muscles which cause stiffness, dental anesthesia, paraesthesias or even hypalgesia, limb trunk and respiratory problems which can contribute to an increase in mortality due to respiratory arrest and muscle atrophy and cognitive involvement causing abnormalities in visual attention and reaction time [12].

Current treatment for MG includes anti-acetylcholinesterase (pyridostigmine) for daily or chronic symptom control, immunomodulatory therapies (intravenous immunoglobulin [IVIG] and plasma exchange), which are typically used for acute exacerbation of disease but have also been used for chronic symptom control and immunosuppressant medications (steroids, azathioprine, cyclosporine, mycophenolate, and methotrexate), which are used for maintenance therapy and typically take weeks to months for effects to be seen [2,13].

Recently, it has been documented in some papers that the use eculizumab, rituximab rozanolixizumab, efgartigimod, monarsen and ultimately a thymectomy can be a possible treatment to MG [13].

With treatment, most individuals with myasthenia can significantly improve their muscle weakness and lead normal or nearly normal lives. In some cases, myasthenia gravis may go into remission, either temporarily or permanently, and muscle weakness may disappear completely making the continuation of the medication unnecessary [14].

In our case, we present a patient with symptoms of weakness, dysphagia and dysarthria who started to deteriorate rapidly to a point in which the doctors thought that he would become quadriplegic since he had miniscule power in his muscles and needed an NG tube for feeding. Furthermore, he had respiratory distress which could have ultimately lead to respiratory arrest potentially causing death.

Fortunately, with the nerve conduction tests and the EMG the doctors could conclude a possible diagnosis of myasthenia gravis which was later confirmed by a positive serum anti-Ach receptor antibodies titer leading to a prompt treatment.

His laboratory test showing low cortisol levels and TSH with high levels of T3 and T4, were a novelty for the doctors and after a thorough researched it was found that the patient was suffering from a condition called autoimmune polyglandular syndrome.

With the associated myasthenia gravis and his laboratory results, it was determined to be type 2 of autoimmune polyglandular syndrome since the patient's findings suggested Addison's disease and an autoimmune thyroid disease apart from the above mentioned myasthenia gravis (see Table 1).

**Table 1:** Autoimmune polyglandular syndrome types and their major features.

Disease	Characteristics
**APS type 1**	Two of the following: • Autoimmune hypoparathyroidism • Autoimmune adrenal insufficiency • Chronic mucocutaneous candidiasis
**APS type 2**	Autoimmune adrenal insufficiency with one or more of the following: • Autoimmune thyroid disease • Type 1 diabetes mellitus
**APS type 3**	Autoimmune thyroid disease with one or more of autoimmune diseases excluding autoimmune adrenal disease, hypoparathyroidism and mucocutaneous candidiasis
**APS type 4**	Two or more organ specific autoimmune diseases and cannot be classified into any of the above three APS types.

*Table from article [15] *.

APS is a rare immune-endocrine syndrome which involves the occurrence of autoimmune adrenal insufficiency, along with one or more of the following entities [3,5,15].

1. Graves' disease or autoimmune hypothyroidism.

2. Type 1 diabetes mellitus.

3. Primary hypogonadism.

4. Myasthenia gravis.

5. Celiac disease.

Other conditions such as vitiligo, alopecia, serositis and pernicious anaemia occur at a greater frequency in these patients; however, they are not included in the diagnostic criteria [15].

In our case, the patient had vitiligo all over his body which is also a remarkable finding since its common to find in patients with APS, but we did not take it into consideration until further research of literature review illuminated its association with APS.

Thyroid hormone replacement initiated before or along with adrenal steroids can potentiate life threatening Addisonian crisis because of thyroxine increasing the basal metabolic rate and inducing a state of relative cortisol deficiency, so it is important to start thyroxine supplementation after the initial stabilisation phase and to have a close follow-up after starting thyroid supplements [15].

Hence, we think that this case report is very important since it recollects all the information needed for the prompt diagnosis and the corresponding management that physicians need to know for the adequate treatment of the patients suffering from this condition.

## 4 Conclusion

It is well-known that having an autoimmune disease leads to a susceptibility of developing other autoimmune processes thus it is important to always have a high index of suspicion in every case and evaluate every patient carefully in order to prevent future complications and life threating events. Our case highlights the need of careful and detailed examination of autoimmune diseases as our prompt diagnosis and treatment with the appropriate hormonal replacement therapy led to a good outcome and prognosis. We also want to highlight the importance of the awareness of the physicians for this disease, focusing mainly on the developing countries that, like Palestine, don't have many resources and the literature respecting this topic is poor, making the diagnosis of this syndrome more difficult.

## Conflicts of interest

There is no conflict of interest.

## Sources of funding

This research did not receive any specific grant from funding agencies in the public, commercial, or not-for-profit sectors.

## Ethical approval

The study is exempt from ethical approval in our institution.

## Consent

Written informed consent was obtained from the patient for publication of this case report and accompanying images. A copy of the written consent is available for review by the Editor-in-Chief of this journal on request.

## Guarantor

Yousef S. Abuzneid.

## Declaration of competing interest

There is no conflict of interest.
